# Molecular Genetic Testing in Reward Deficiency Syndrome (RDS): Facts and Fiction

**DOI:** 10.17756/jrds.2015-009

**Published:** 2015-03-19

**Authors:** Kenneth Blum, Rajendra D. Badgaiyan, Gozde Agan, James Fratantonio, Thomas Simpatico, Marcelo Febo, Brett C. Haberstick, Andrew Smolen, Mark S. Gold

**Affiliations:** 1 Department of Psychiatry, McKnight Brain Institute, University of Florida, Gainesville, FL, USA; 2 Department of Addiction Research & Therapy, Malibu Beach Recovery Center, Malibu Beach, CA, USA; 3 Divisions of Addiction Services, and Applied Clinical Research, Dominion Diagnostics, LLC, North Kingstown, RI, USA; 4 Department of Psychiatry, Human Integrated Services Unit University of Vermont Center for Clinical & Translational Science, College of Medicine, Burlington, VT, USA; 5 Department of Psychiatry and Division of Neuroimaging, University of Minnesota College of Medicine, MN, USA; 6 Institute for Behavioral Genetics, University of Colorado Boulder, Boulder, CO, USA; 7 Departments of Psychiatry & Behavioral Sciences at the Keck, University of Southern California, School of Medicine, CA, USA; 8 Director of Research, Drug Enforcement Administration (DEA) Educational Foundation, Washington, D.C, USA

**Keywords:** Reward Deficiency Syndrome, Brain Reward Cascade, DRD2, Gene variations, Genetic Addiction Risk Score

## Abstract

**Background:**

The Brain Reward Cascade (BRC) is an interaction of neurotransmitters and their respective genes to control the amount of dopamine released within the brain. Any variations within this pathway, whether genetic or environmental (epigenetic), may result in addictive behaviors or RDS, which was coined to define addictive behaviors and their genetic components.

**Methods:**

To carry out this review we searched a number of important databases including: Filtered: Cochrane Systematic reviews; DARE; Pubmed Central Clinical Quaries; National Guideline Clearinghouse and unfiltered resources: PsychINFO; ACP PIER; PsychSage; Pubmed/Medline. The major search terms included: dopamine agonist therapy for Addiction; dopamine agonist therapy for Reward dependence; dopamine antagonistic therapy for addiction; dopamine antagonistic therapy for reward dependence and neurogenetics of RDS.

**Results:**

While there are many studies claiming a genetic association with RDS behavior, not all are scientifically accurate.

**Conclusion:**

Albeit our bias, this Clinical Pearl discusses the facts and fictions behind molecular genetic testing in RDS and the significance behind the development of the Genetic Addiction Risk Score (GARS_PREDX_™), the first test to accurately predict one’s genetic risk for RDS.

## Introduction

In 1990 Blum’s laboratory at the University of Texas along with Ernest Nobles’ group at UCLA discovered the first genetic association with severe alcoholism, the Dopamine D2 receptor gene located on chromosome 11 q22-q23 [1]. This seminal work was published in the prestigious Journal of the American Medical Association (JAMA) [2]. The article was fraught with controversy from the scientific community [3] but now almost a quarter of a century later it has been globally confirmed, and it is considered a major gene involved in all addictive behaviors (PUBMED 3-1-15, 3864 searches) [4].

### Search Information

To carry out this review we searched a number of important databases including: Filtered: Cochrane Systematic reviews; DARE; Pubmed Central Clinical Quaries; National Guideline Clearinghouse and unfiltered resources: PsychINFO; ACP PIER; PsychSage; Pubmed/Medline. The major search terms included: dopamine agonist therapy for Addiction; dopamine agonist therapy for Reward dependence; dopamine antagonistic therapy for addiction; dopamine antagonistic therapy for reward dependence. Our results produced the following: dopamine agonistic therapy for addiction-Cochrane Systematic reviews-o; DARE-0; Pubmed Central Clinical Quaries-9, National Guideline Clearinghouse-0; PsychINFO-0; ACP PIER-83; PsychSage-15; Pubmed/Medline-501; dopamine agonist for addiction-Cochrane Systematic reviews-3; DARE-3; Pubmed Central Clinical Quaries-10; National Guideline Clearinghouse-0; ACP PIER-0; Psychsage-15; Pubmed/Medline-13; dopamine agonistic therapy for reward dependence-Cochrane Systematic reviews-0; DARE-0; Pubmed Central Clinical Quaries-1; National Guideline Clearinghouse-0; PsychINFO-0; ACP PIER-0, PsychSage-0; Pubmed/Medline-62; dopamine agonist for reward dependence- Cochrane Systematic reviews-0; DARE-0; Pubmed Central Clinical Quaries-337; National Guideline Clearinghouse-0; PsychINFO-1; ACP PIER-0; PsychSage-0; Pubmed/Medline-120; dopamine antagonistic therapy for addiction-Cochrane Systematic reviews-0; DARE-0; Pubmed Central Clinical Quaries-0; National Guideline Clearinghouse-0; PsychINFO-0; ACP PIER-0; PsychSage-0; Pubmed/Medline-633. Clearly we utilized a combination of Pubmed Central Clinical Quaries and Pubmed/Medline for our reliable review search as well as author searches based on personal knowledge of the field. In terms of neurogenetics we utilized PUBMED primarily.

### Examples of Neurogenetics

Clark et al. [4] analyzed the role of rs1076560 in opioid dependence by genotyping 1,325 opioid addicts. rs1076560 was found to be nominally associated with opioid dependence. However, when both opioid-addicted ancestral samples were combined, rs1076560 was significantly associated with increased risk for drug dependence (p = 0.0038, OR = 1.29). Other examples include the work of David’s group [5] and Lerman et al. [6] showing the association of both the dopamine D2 transporter gene polymorphism as well as polymorphisms of the DRD2 with nicotine addiction. [7, 8, 9] Gilbert et al. [10] and Spitz et al. [11] found dopaminergic gene polymorphisms with abstinence from smoking.

We believe these previous studies [1–3] laid down the foundation for the subsequent development of the field “Psychiatric Genetics”. As expected, we now know following thousands (15,074) of peer reviewed articles that all addictive behaviors involve polygenic variants including many single nucleotide polymorphisms (SNPs) and even point-mutations such as the GABA (A) receptor subtypes [12]. As a follow up to the original study one of us (KB) coined the term Reward Deficiency Syndrome (RDS) to help define not only drug, alcohol, food, and behavioral addictions like gambling, sex etc. but to understand the relationship of genetic risk [13].

### Can we Predict Risk Using Genetic Testing?

Adopting a Bayesian approach, earlier studies from Blum’s laboratory determined a Positive Predictive Value (PPV) for the DRD2 A1 variant (low number of D2 receptors) of 74%, indicating that if a child is born with this polymorphism they have a very high risk of becoming addicted to either drugs, food, or aberrant behaviors at some point in their future [14, 15].

Over the many years to come since the 1990 finding, laboratories all across the globe including NIDA and NIAAA not only confirmed this early work [2] but extended the magnitude of many other candidate genes, especially genes and second messengers located in the reward circuitry of the brain [16]. Specifically, Moeller et al. [17] suggested that drug cues contribute to relapse, and their neurogenetic results have identified the DAT1R 9R-allele as a vulnerability allele for relapse especially during early abstinence (e.g., detoxification). The DAT1 9 allele influences the fast acting transport of dopamine sequestered from the synapse leading to a hypodopaminergic trait. Along these lines in conjunction with one of us (KB) and Gerald Kozlowski they developed the “Brain Reward Cascade”(BRC) [18]. This concept served as a blue print for how neurotransmitters interact in the reward system of the brain. In addition, it has been firmly established that respective reward genes that regulate these chemical messengers ultimately control the amount of dopamine released into not only the reward site but other regions of the brain. Moreover, it is well established that resting state functional connectivity integrity is important for normal homeostatic functioning. Zhang et al. [19] recently showed that in heroin addicts there is reduced connectivity between dorsal anterior cingulate cortex (dACC) and rostral (rACC), as well as reduced connectivity between subcallosal (sACC) and dACC. Their findings of variations of functional connectivity in three sub-regions of ACC in heroin addicts implied that these sub-regions of the ACC together with other key brain areas (such as dorsal striatum, putamen, orbital frontal cortex, dorsal striatum, cerebellum, amygdale, etc.) potentially play important roles in heroin addiction. Most recently Blum’s laboratory along with Zhang’s group [20] in abstinent heroin addicts showed that KB220Z™ a complex putative dopamine D2 agonist, induced an increase in BOLD activation in caudate-accumbens-dopaminergic pathways compared to placebo following one-hour acute administration. Furthermore, KB220Z™ also reduced resting state activity in the putamen of abstinent heroin addicts. In the second phase of this pilot study of all ten abstinent heroin-dependent subjects, three brain regions of interest (ROIs) we observed to be significantly activated from resting state by KB220Z compared to placebo (P < 0.05). Increased functional connectivity was observed in a putative network that included the dorsal anterior cingulate, medial frontal gyrus, nucleus accumbens, posterior cingulate, occipital cortical areas and cerebellum.

Importantly, Blum’s laboratory proposed [21] that any disturbance along this brain reward cascade due to either gene variations (polymorphisms) or environment (epigenetics) will result in aberrant addictive behaviors or RDS. In spite of a global search to uncover specific or candidate genes or even clusters of genes characterized from high-density SNP arrays, it is well-known that many attempts have not replicated or been inconclusive. However, Palmer et al. [22] recently showed that between 25–36 percent of the variance in the generalized vulnerability to substance dependence is attributable to common single nucleotide polymorphisms. Moreover, the additive effect of common single nucleotide polymorphisms is shared across important indicators of comorbid drug problems. Furthermore, as a result of these studies more recent evidence has revealed that specific candidate gene variants account for risk prediction. Recently, two independent studies reveal potential candidates such as synuclein alpha (SNCA) [23] and the human-specific isoform of the voltage-gated sodium channel subunit SCN4B [24]. However, others could not find any association with a number of candidate genes instead found that family issues were a better predictor [25].

### Fiction

While there is a plethora of very positive experiments involving thousands of studies for many candidate gene associations with all RDS behaviors, there are also negative results [26]. Currently, a number of companies have entered the genetic testing arena in the addiction and pain industrial space claiming “personalized care”. However, we believe these companies have not done their homework in a scientific manner. These issues include exaggerated claims such as using Blum’s original work [13, 14] stating that their genetic test is 74% predictive. This is indeed false because they use one gene (DRD2) to back their claim and commercialize a full panel of other candidate genes and never carried out any outcome studies with their panel. Additionally, they make other false claims suggesting that patient’s results are compared to population controls. Review of their so called “disease free” controls reveal significant flaws especially in light of not controlling for a remarkable list of RDS behaviors [27]. They would have to utilize what has been termed “Super-Controls.” Simply stated population controls may carry many invisible RDS behaviors that must be identified so that the control would be RDS free.

Otherwise utilization will lead to spurious and false results [28]. Another issue is that these companies have selected genes that may be involved in risky behavior but they do not utilize the correct variant in their tests or use very rare variants that do not truly prove addiction risk. Specifically, Mayer and Höllt [29] correctly proposed that “the vast number of non-coding, intronic or promoter polymorphisms in the opioid receptors may influence addictive behavior, but these polymorphisms are far less studied, and their physiological significance remains to be demonstrated.” Most importantly, these companies have never performed research to show whether their genetic full-panel test significantly predicts anything let alone addiction risk or any associated behaviors.

### Facts

While we the authors may have a personal bias because over the many years that Blum’s laboratory has dedicated work to develop an accurate genetic test to predict true liability/risk for RDS and associated behaviors, we will attempt to explain why our current laboratory has successfully developed the first Genetic Addiction Risk Score (GARS_PREDX_™) in conjunction with the Institute of Behavioral Genetics, University of Colorado, Boulder.

To develop GARS_PREDX_™ we first selected ten reward candidate genes and a number of SNPs and point mutations that influence the net release of dopamine at the brain reward site. The variants or SNPs, including point-mutations, were chosen to reflect a hypodopaminergic trait. In terms of validation we partnered with the developers of the Addiction Severity Index-Media Version (ASI-MV), a test mandated in 13 states, for both alcohol and drug severity risk scores [30].

We contacted eight very diverse treatment centers across the United States resulting in a total of 393 subjects that were genotyped using the selected GARS_PREDX_™ panel. All the data was genotyped and analyzed at the Institute for Behavioral Genetics (IBG) at the University of Colorado Boulder. Without going into specifics we found a significant association between a summed score of all GARS panel risk alleles (variant forms) and both the ASI-MV alcohol and drug severity indices in a total of 273 subjects.

In fact, the higher the number of risk alleles the stronger the prediction of alcohol or drug use severity. It was also found that family problems, psychological issues and medicalization significantly correlated as well. One important caveat was that if we changed any specific SNP the significance was lost. This strongly suggests how important the selected GARS_PREDX_™ panel is and any deviation will produce false results that may occur with other commercial tests that have no research to validate their tests. A full length paper on these results will be published elsewhere whereby all gene polymorphisms will be displayed.

## Conclusion

In summary, based on this seminal research and unlike other existing genetic tests we are poised to launch the first proven and validated Genetic Addiction Risk Score that will have important clinical benefits including personalized medicine and assessment of RDS risk. The test will also include the P450 system of genetic variants that influence how individuals metabolize opioids [31]. Clinically, the future is here and the treatment of chronic addiction and pain depends on scientifically sound appropriate early genetic risk diagnosis leading to real personalized (not fiction) care of the patient.
